# Physical Activity, Sleep, and Sedentary Behavior among Successful Long-Term Weight Loss Maintainers: Findings from a U.S. National Study

**DOI:** 10.3390/ijerph18115557

**Published:** 2021-05-22

**Authors:** Gregory Knell, Qing Li, Elisa Morales-Marroquin, Jeffrey Drope, Kelley Pettee Gabriel, Kerem Shuval

**Affiliations:** 1 Department of Epidemiology, Human Genetics and Environmental Sciences, The University of Texas Health Science Center at Houston (UTHealth), Houston, TX 77030, USA; flor.e.moralesmarroquin@uth.tmc.edu; 2 Center for Pediatric Population Health, The University of Texas Health Science Center at Houston (UTHealth), Dallas, TX 75390, USA; 3 Children’s Health Andrews Institute for Orthopaedics and Sports Medicine, Plano, TX 75024, USA; 4 Department of Intramural Research, American Cancer Society, Atlanta, GA 30303, USA; qing.li@cancer.org; 5 Department of Health Policy and Administration, School of Public Health, University of Illinois at Chicago, Chicago, IL 60608, USA; jdrope@uic.edu; 6 Department of Epidemiology, School of Public Health, University of Alabama at Birmingham, Birmingham, AL 35294, USA; gabrielk@uab.edu; 7 The Cooper Institute, Dallas, TX 75230, USA; kshuval@cooperinst.org; 8 Department of Epidemiology, School of Public Health, University of Haifa, Haifa 3498838, Israel

**Keywords:** weight loss, exercise, sedentary behavior, sleep, accelerometry

## Abstract

Despite adults’ desire to reduce body mass (weight) for numerous health benefits, few are able to successfully lose at least 5% of their starting weight. There is evidence on the independent associations of physical activity, sedentary behaviors, and sleep with weight loss; however, this study provided insight on the combined effects of these behaviors on long-term body weight loss success. Hence, the purpose of this cross-sectional study was to evaluate the joint relations of sleep, physical activity, and sedentary behaviors with successful long-term weight loss. Data are from the 2005–2006 wave of the National Health and Examination Survey (NHANES). Physical activity and sedentary behavior were measured with an accelerometer, whereas sleep time was self-reported. Physical activity and sleep were dichotomized into meeting guidelines (active/not active, ideal sleep/short sleep), and sedentary time was categorized into prolonged sedentary time (4th quartile) compared to low sedentary time (1st–3rd quartiles). The dichotomized behaviors were combined to form 12 unique behavioral combinations. Two-step multivariable regression models were used to determine the associations between the behavioral combinations with (1) long-term weight loss success (≥5% body mass reduction for ≥12-months) and (2) the amount of body mass reduction among those who were successful. After adjustment for relevant factors, there were no significant associations between any of the independent body weight loss behaviors (physical activity, sedentary time, and sleep) and successful long-term weight loss. However, after combining the behaviors, those who were active (≥150 min MVPA weekly), regardless of their sedentary time, were significantly (*p* < 0.05) more likely to have long-term weight loss success compared to the inactive and sedentary referent group. These results should be confirmed in longitudinal analyses, including investigation of characteristics of waking (type, domain, and context) and sleep (quality metrics) behaviors for their association with long-term weight loss success.

## 1. Introduction

Nearly two-thirds of overweight or obese adults indicate their desire to reduce body mass (weight), and around 40% of those are able to lose at least 5% of their starting weight in the short term [1]. However, very few are able to successfully maintain weight loss over a meaningful period of time (≥12 months) [1,2,3,4,5]. Given the health benefits conferred by long-term weight loss [3,4,6], it is important to identify strategies employed by those successfully attaining this goal in the long term to optimize behavioral weight loss programs [7].

Weight loss and weight loss maintenance are mostly dependent, beyond genetics, on the energy balance equation, such that those that are able to lose and maintain weight loss have a negative energy balance [8,9,10]. On the energy intake side of the equation, findings from the National Weight Control Registry indicate that individuals who are successful at losing weight and maintaining weight loss over time utilize behavior strategies including consuming a low-fat diet and self-monitoring food intake [11,12,13]. Furthermore, successful weight loss maintainers aim to control total energy intake by drinking water and minimizing consumption of sugar-sweetened beverages coupled with habitually engaging in physical activity [11,13,14].

Physical activity, along with sleep and sedentary time, make up the three physical behaviors on the energy expenditure side of the equation [15,16]. Each of these has been studied for its independent associations with weight loss. Regarding physical activity, initiating a structured exercise program and sustaining engagement has been cited as a key component of weight loss maintenance [10,17]. Additionally, it has been found that replacing sedentary time (activities requiring ≤1.5 metabolic equivalents (METs) [18]), with moderate- to vigorous-intensity physical activity (MVPA) predicts weight change [15,19,20]. Less evidence exists on the relationship between sedentary behavior and weight loss maintenance [21,22]; however, higher sedentary time has been associated with weight regain [19,23,24]. With regards to sleep, evidence has shown that successful weight loss maintainers sleep on average one hour longer than their less successful counterparts and maintain a regular sleep schedule [21,25,26].

Despite the independent findings relating these three behaviors with successful long-term weight loss maintenance, it is unclear if their co-occurrence is related to long-term weight loss maintenance. The various combinations of these behaviors may be useful in developing a more sophisticated understanding of how their co-action may relate to long-term weight loss. From a public health perspective, these results may be helpful in generating hypotheses to continue exploring the effects of lifestyle behaviors on weight loss. Therefore, the purpose of this study was to evaluate the association between combinations of physical activity, sedentarism, and sleep with successful long-term weight loss among a population-based sample of United States (US) adults.

## 2. Materials and Methods

### 2.1. Study Design, Setting, and Participants

Data for this cross-sectional study were retrieved from the 2005–2006 National Health and Examination Survey (NHANES), which collects cross-sectional health and nutrition information biannually using a complex sampling design to produce a nationally representative sample of US residents [27]. The 2005–2006 administration of NHANES was utilized due to it being the most recent administration with accelerometer-measured physical activity and sedentary behavior available at the time of analysis [28].

For the current study, inclusion criteria comprising adult accelerometer participants (*n* = 6849), who (1) had worn an accelerometer for more than 4 days with at least 10 h per day (*n* = 6282), (2) were aged 18–65 years (*n* = 3262), and (3) were at one point during their lifetime overweight, i.e., “historic” BMI ≥ 25 kg/m^2^ (*n* = 2191). Participants who were underweight (BMI < 18.5 kg/m^2^) the year of the measurement or in the previous year were excluded (*n* = 4) due to the potential for underlying medical conditions, as well as those who were pregnant (*n* = 163). A complete case analysis approach was used, resulting in a final analytic sample of 1735.

### 2.2. Data Collection

NHANES collects participant data through an at-home visit (unweighted *n* = 10,348 (80.5% response rate)) and at a mobile examination center among those agreeing to a physical examination (unweighted *n* = 9950 (77.4% response rate)) [27]. Data collected at the at-home visit included a self-reported questionnaire conducted by a trained data collector (interviewer). Data collected at the physical exam include blood serum samples for various laboratory testing, and various other physiological measurements and medical examinations. For the current analyses, all data other than physical activity, sedentary, and smoking measures were obtained through the questionnaire. Participants aged 6 and older were asked to wear a hip-worn accelerometer during waking hours over a seven-day observation period. Participants were asked to wear the monitor for seven consecutive days and to remove it at bedtime and before swimming or bathing (as monitors were not water proof) [29]. The National Center for Health Statistics ethics review committee approved all data collection protocols and received informed consent from all participants. The NHANES data, methods, and procedures are explained in greater detail elsewhere [27].

### 2.3. Measures

The primary independent variables were behavioral combinations of physical activity, sedentary time, and sleep. Physical activity and sedentary time were measured via ActiGraph AM-7164 (ActiGraph, Pensacola, FL, USA) accelerometers worn on the right hip [30]. The accelerometers measure the intensity of physical movement in a single plane (uniaxial) in one-minute intervals (epochs) [31]. The epochs are then processed into counts per minute (cpm) [31]. CPM-specific thresholds are used to classify movement by intensity of physical activity: sedentary (<100 cpm), light intensity (100–1951 cpm), moderate to vigorous intensity (≥1952 cpm) [32]. For each participant, the average time spent in each of these intensity categories was summed and averaged over the observation period. Participants were classified as “active” if accumulating ≥150 min per week of accelerometer-derived moderate- to vigorous-intensity physical activity (MVPA), as is consistent with the 2018 Physical Activity Guidelines for Americans [33]. Given the absence of a specific daily threshold for sedentary time in the literature [34,35], this variable was categorized into quartiles and then used to estimate the multivariable associations with long-term weight loss. These results demonstrate that the 1st, 2nd, and 3rd quartiles were each independently associated with long-term weight loss success compared to the 4th quartile (i.e., highest levels of sedentary time). Therefore, sedentary time was classified as high or low based on the 4th quartile vs. 1st–3rd quartiles. The accelerometer data collected in NHANES have been examined widely to understand the relation between physical movement and various health outcomes and have been demonstrated to be valid estimates of physical movement [29,30].

Sleep time was estimated as part of a larger, validated sleep questionnaire (Functional Outcomes of Sleep Questionnaire [36,37]), since the accelerometer was only worn during waking hours. The reported average sleep time per night was dichotomized into “ideal sleepers” (≥7 h of sleep per night) and “short sleepers” (<7 h of sleep per night) [38]. From the independent subgroups (i.e., active vs. inactive, high vs. low sedentary time, and ideal vs. short sleep), twelve categories were created based on all possible combinations.

The dependent variable was long-term weight loss success. Participants’ self-reported body mass was estimated at three time points: current body mass, body mass one year prior, and historical maximum body mass. The higher reported body mass between current body mass or body mass one year prior was subtracted from historical maximum body mass and then divided by the historical maximum body mass [6]. This calculated value was multiplied by 100 to determine the participant’s percent of body mass reduction. Those achieving at least 5% were classified as maintaining successful long-term weight loss [6]. Self-reported body mass using more recent (2005–2008) NHANES data has been found to be more accurate than previous NHANES data (1988–1994) [39], and historical reporting of body mass (1 year prior and historical maximum body mass) has been found to be minimally biased [40].

### 2.4. Statistical Analysis

Descriptive statistics and the bivariable relations of participant characteristics and summary estimates of physical activity, sedentary behavior, and sleep by long-term weight loss success were analyzed using *t*-tests, median tests for differences, or chi square tests as appropriate. The multivariable associations among independent estimates of physical activity, sedentary time, and sleep with long-term weight loss success were analyzed using logistic regression models. Two-step models were used to evaluate the association of combinations of weight-loss-related physical behaviors (sleep, physical activity, and sedentary behaviors) with successful long-term weight loss. The first part of the two-step model used a logit model to determine if there was an association between the behavioral combination and any amount of long-term weight loss success greater than 0%. The second part of the two-step model only included those who were successful at losing at least some weight loss (i.e., >0%) and then estimated the amount of weight loss associated with the behavioral combination using a generalized linear model (GLM), where Gaussian family and log link function were employed due to the modified park test (chi2 = 28.24; *p* = 0.00) and box Cox test (θ = 0.08; *p* = 0.004) results.

All models were adjusted for daily caloric intake (kcal), age (continuous, years), sex, race/ethnicity (Hispanic, non-Hispanic white, non-Hispanic black, other), marital status (married/not married), education (not college graduate/college graduate), annual household income (< USD 20,000; USD 20,000–44,999; USD 45,000–74,999; ≥ USD 75,000), household size (continuous), current smoking (yes/no, cutoff point for a cotinine level of 3.08 ng/mL) [41], self-rated health status (poor, fair, good, very good, excellent), and accelerometer wear time for sedentary time and physical activity. Statistical analysis was performed using StataSE 15.1 (StataCorp LLC, College Station, TX, USA), while employing appropriate survey weights to enable representativeness; analyses were performed February–April 2020. This study received exempt status from the University of Texas Health Science Center at Houston (UTHealth) institutional review board (IRB# HSC-SPH-17-0925).

## 3. Results

Participant characteristics are shown in Table 1. A total of 525 participants (30.26%) successfully maintained at least 5% weight loss for 12 months. When comparing participant characteristics by long-term weight loss success, there were significant differences (*p* < 0.05) by age, self-reported health status (good), and household income (USD 20,000–44,999 annual).

Participants’ sleep, sedentary time, and physical activity levels are described by long-term weight loss success using bivariable analyses in Table 2. A greater proportion of unsuccessful long-term weight loss maintainers slept the ideal number of hours (≥7 h per night) than those successfully maintaining long-term weight loss; however, these differences were not statistically significant (*p* = 0.35). Additionally, those successfully maintaining long-term weight loss spent less time sedentary (*p* = 0.36), and more time in light-intensity physical activity (*p* = 0.84) and MVPA (*p* = 0.55); however, these differences were not statistically significant.

The multivariable relationship between long-term weight loss success and each of the independent behaviors (sleep, sedentary, and physical activity) is presented in Table 3. None of the behaviors were significantly associated with long-term weight success (*p* > 0.05); however, compared to the reference, lower levels of sedentary time were associated with higher odds of long-term weight loss success.

The results from the two-step models are depicted in Table 4. The logit portion (step one) of the model indicates the behavioral combination’s association with any amount (greater than 0%) of long-term weight loss success. In step one, there were significant associations found for the *inactive + low sedentary* (OR [95% CI] = 1.61 [1.12–2.31], *p* < 0.01), *active + high sedentary* (OR [95% CI] = 1.61 [1.10–2.38], *p* < 0.02), and *active + low sedentary* (OR [95% CI] = 1.55 [1.05–2.28], *p* < 0.03) behavioral combination after adjusting for covariates. The *active* portion of the behavioral combination describes an individual that is sufficiently physically active to meet the minimum recommendations for physical activity (150 min per day of MVPA), while the *low sedentary* portion describes an individual averaging less than 3592 min per day of sedentary time. In the GLM portion of the model, the analysis only includes those that were successful at long-term weight loss (>0%) and estimates the amount or degree of weight loss associated with each modeled behavioral combination. Among successful long-term weight loss maintainers, compared to the *short sleepers + inactive* combination, the *ideal sleepers + active* combination was significantly associated (*p* = 0.01) with a decrease in weight loss after accounting for relevant factors.

## 4. Discussion

Taken together, of all the elements of the 24 h activity behavioral combinations (physical activity, sleep, sedentary time), physical activity and sedentary time together was the most consistently associated with long-term weight loss success. Notably, physical activity was not independently associated with long-term weight loss success but required the combination with sedentary time. While these findings were statistically significant, they pertained to any level of weight loss (>0%) and were not clinically meaningful. That is, when it came to clinically meaningful weight loss (≥5%) [7], these relationships were not significant. These results warrant future research with a larger sample size, improved measure, and longitudinal design.

Previous research has found each of the activity and sleep behaviors to be independently associated with weight loss and long-term weight loss maintenance. Individuals successfully maintaining weight have reported greater levels of MVPA than those who are unsuccessful [42]. Objective measures of energy expenditure have recently confirmed these findings by indicating that, compared to normal weight and overweight/obese controls, individuals that have successfully maintained long-term weight loss have markedly higher levels of physical-activity-related energy expenditure [43]. Research on sedentary behavior has demonstrated that successful weight loss maintainers have lower levels of sedentary time in comparison to controls [21,22]. Additionally, sedentary time has been found to be associated with weight regain [19,23,24]. Finally, sleep research has shown that, amongst successful weight loss maintainers, those who experience more success sleep on average 1 h longer than their less successful counterparts [25]. Not only has sleep duration been shown to have health effects but also sleep quality (efficiency, staying asleep), architecture (sleep stages), timing (bedtime/wake up time), consistency (day to day variability), and continuity (variability in sleep duration within the same night) [44,45]. Future research should aim to evaluate these additional sleep metrics for their relationship with other behaviors in the activity and sleep cycle and related health outcomes.

Despite these findings in the literature, the current study found no association between weight loss and the activity and sleep behaviors independently, but rather only as combined behaviors (physical activity and sedentary time). It is of note that the model with interactions did not include the main effects of variables in interaction terms (see Table 4). Subsequently, we computed models with interactions and the main effects of interaction terms, which appear in the Appendix; findings did not change materially. As others have pointed out, the combined effect of these behaviors may be more important to health than any one behavior independently [46]. However, the results of the current study did not entirely support this notion. We found that only the combined behaviors of physical activity and sedentary time were related to weight loss success, but neither this combination nor the other behavioral combinations were consistently related to the degree of weight loss among those who had successfully lost weight. Additionally, many of the coefficients relating the degree of weight loss (step two in the two-step model) were negative values, indicating a negative association between the behavioral combinations and degree of weight loss. The lack of strength and consistency in these findings may be an indication of potential role diet plays in weight loss success and maintenance; as stated in the 2018 Physical Activity Guidelines for Americans, the “health benefits of physical activity are generally independent of body weight” [33]. From a clinical perspective, these results should not be interpreted to mean that physical activity confers no weight-loss-related benefits, but rather that “the amount of physical activity necessary to achieve [weight loss] varies greatly from person to person” [33] and should be considered a tool for weight maintenance rather than weight loss. Given that the stated objective of this study was to determine the associations between physical behaviors related to energy expenditure (or lack thereof) and weight loss, we only adjusted for caloric intake as a covariate. Given these findings, and the known importance of diet quality and caloric intake within the weight loss paradigm, future studies should aim to determine the combined effect of physical activity, sleep, sedentary time, and diet on long-term weight loss success and maintenance. Furthermore, healthcare providers should continue to recommend and advise on strategies to engage in a healthy lifestyle that includes a comprehensive, multicomponent physical activity [47] and nutrition [48] plan for health benefits (including weight loss maintenance).

This study is not without limitations. First, the study design was cross-sectional and, therefore, precludes establishing a temporal relationship between the behavioral combinations and long-term weight loss success and subsequently reaching causal inferences. However, the weight loss estimates were based on historical maximum body mass, thereby allowing the estimation of change in body mass over time. Meanwhile, the activity and sleep behavior estimates occurred at the time of data collection and, therefore, allow one to assume that these are the behavioral combinations of individuals currently successfully maintaining long-term weight loss. Furthermore, these data from 2005–2006 may be considered by some as outdated. Although the NHANES has more recent accelerometer data available (collected between 2011–2012 and 2013–2014), these data are not provided in such a format to allow the analysis as was done in the current study. Therefore, the current accelerometry data (NHANES 2005–2006) are the most accurate and latest assessment of physical activity among a nationally representative sample of Americans. Second, the estimates for physical activity and sleep were categorized into clinically meaningful categories; however, in doing so, the thresholds used to construct the categories may not be relevant to the outcome under study, and the use of categorical data in this way may limit the statistical power to detect an association. Moreover, the study relies on self-reported sleep, height, and body mass, which could lead to under-reporting (body mass) or over-reporting (height). However, since these variables were measured using a standard protocol, this likely leads to non-differential misclassification [49]. It should also be noted that the use of body mass reduction may not reflect a reduction in fat mass, but rather a reduction in some other component of body composition (fat-free mass, bone mineral density). Unfortunately, historical body composition data are not available in the NHANES data, which would be required to estimate changes in body composition over time as done here. Finally, the sample size used in this study is relatively small, particularly when considering the study’s aim of examining joint relationships of lifestyle behavior and weight loss.

## 5. Conclusions

The current study was based on a representative sample of US adults that utilized device-based measures of physical activity and sedentary behavior, combined with self-reported sleep duration to assess the relation between combinations of physical activity, sleep, and sedentary behaviors in individuals with long-term weight loss maintenance. The results indicate that after accounting for covariates, the combined association of physical activity and limited sedentary time is related to weight loss but without clinical significance. Specifically, these behaviors did not appear to have consistent and meaningful relations with the degree of weight loss. Future longitudinal research using larger sample sizes with objectively measured sleep should confirm these findings, while further examining the elements of each of these behaviors and related health outcomes.

## Figures and Tables

**Table 1:** Participants’ characteristics by long-term weight loss success.

	Long-Term Weight Loss Success ^a^ % (95% CI)		
Characteristic	Unsuccessful (<5%)	Successful (≥5%)	*p*
Age (years), means (SE)			
Current	42.8 (0.7)	44.8 (0.5)	0.01
Sex			
Female	46.5 (43.5–49.5)	42.8 (39.3–48.5)	0.17
Male	53.5 (50.5–56.5)	57.2 (51.9–62.6)	0.17
Current smoking			
Smoker	27.9 (23.5–32.3)	31.5 (25.6–37.4)	0.25
Nonsmoker	72.1 (67.7–76.5)	68.5 (62.6–74.4)	0.25
Household size			
Count, mean (SE)	3.2 (0.1)	3.0 (0.1)	0.10
Race/ethnicity			
White, non-Hispanic	69.1 (62.2–76.0)	72.8 (65.9–79.8)	0.12
Black, non-Hispanic	13.8 (8.7–18.8)	11.5 (6.0–17.1)	0.19
Hispanic	12.6 (8.8–16.4)	11.2 (7.2–15.2)	0.40
Other ^b^	4.5 (1.6–7.4)	4.5 (2.5–6.5)	0.99
College graduate			
No	76.0 (70.6–81.4)	73.4 (63.6–83.3)	0.53
Yes	24.0 (18.6–29.4)	26.6 (16.7–36.5)	0.53
Marital status			
Not married	30.3 (25.9–34.8)	32.5 (26.0–39.0)	0.47
College graduate or more	69.7 (65.2–74.1)	67.5 (61.0–74.0)	0.47
Self-report health status			
Excellent	7.7 (6.0–9.5)	10.4 (6.9–13.9)	0.92
Very good	34.8 (31.4–38.2)	34.0 (29.8–38.3)	0.20
Good	41.5 (38.7–44.4)	36.8 (31.0–42.6)	0.03
Fair	13.9 (11.7–16.1)	16.7 (12.3–21.0)	0.62
Poor	2.0 (1.2–2.9)	2.1 (0.5–3.7)	0.05
Annual household income			
below USD 20,000	12.2 (10.0–14.5)	12.2 (9.8–14.6)	0.98
USD 20,000–44,999	24.5 (19.2–29.7)	24.2 (18.1–30.3)	0.94
USD 45,000–74,999	26.7 (23.7–29.6)	31.6 (26.5–36.4)	0.04
USD 75,000 or more	36.7 (31.1–42.2)	32.0 (24.1–39.9)	0.30
Daily caloric intake (kcal d^−1^) mean (SE)	2327.2 (27.8)	2325.2 (51.02)	0.97

Abbreviations: CI, confidence interval; kcal d^−1^, SE, standard error. Notes: ^a^ long-term weight loss was calculated by taking the participants’ reported body mass 1 year ago or current body mass (the higher of the two), subtracting that figure from their historical maximum body mass, and then dividing by their historical maximum body mass and multiplying by 100. ^b^ Includes Asian, Native Hawaiian/Pacific Islander, and non-Hispanic multiple race/ethnicities.

**Table 2:** Comparison of estimates of sleep, sedentary time, and physical activity by long-term weight loss groups.

	Long-Term Weight Loss Success ^a^		
	Unsuccessful (<5%)	Successful (≥5%)	*p*
Sleep			
Total sleep time (h d^−1^), mean (SE)	6.8 (0.04)	6.7 (0.08)	0.46
Ideal sleepers ^b^	62.8 (59.4–66.3)	59.3 (52.3–66.2)	0.35
Sedentary			
Total sedentary time (min d^−1^), mean (SE)	2878.5 (40.9)	2817.1 (55.9)	0.36
High sedentary ^c^	23.1 (20–6-25.6)	19.4 (14.5–24.2)	0.13
Physical activity			
Light physical activity (min d^−1^), mean (SE)	1527.4 (27.2)	1535.6 (37.4)	0.84
MVPA (min d^−1^), mean (SE)	144.1 (6.8)	148.4 (8.2)	0.55
Total accelerometer wear time (minutes), mean (SE)	5111.3 (71.4)	5060.9 (72.9)	0.47
Accelerometer counts (ct/min d^−1^), mean (SE)	47.0 (0.8)	47.9 (0.9)	0.44
Active ^d^, % (95% CI)	38.6 (33.1–44.1)	37.5 (31.4–43.7)	0.75

Abbreviations: CI, confidence interval; h d^−1^, hours per day; min d^−1^, minutes per day; MVPA, moderate- to vigorous-intensity physical activity; SE, standard error. Notes: ^a^ Long-term weight loss was calculated by taking the participants’ reported body mass 1 year ago or current body mass (the higher of the two), subtracting that figure from their historical maximum body mass, and then dividing by their historical maximum body mass and multiplying by 100. ^b^ Ideal sleepers are those that report sleeping for ≥7 h per night. ^c^ High sedentary are those in the 4th quartile of sedentary time per day. ^d^ Active are those that accumulate greater than or equal to 150 min per week of moderate to vigorous aerobic physical activity.

**Table 3:** Multivariable associations between sleep, sedentary time, and physical activity with long-term weight loss success.

	Long-Term Weight Loss Success ^a^		
	OR	95% CI	*p*
Sleep ^b^			
Poor sleepers	1.0 (ref.)		
Ideal sleepers	0.83	0.60–1.16	0.25
Sedentary			
4th quartile	1.0 (ref.)		
1st quartile	1.57	0.63–3.90	0.30
2nd quartile	1.55	0.90–2.66	0.11
3rd quartile	1.29	0.80–2.09	0.28
Physical activity			
Inactive ^c^	1.0 (ref.)		
Active ^d^	1.01	0.76–1.36	0.92

Abbreviations: CI, confidence interval; Coef, coefficient; OR, odds ratio; P, *p*-value; Ref, reference group. Notes: ^a^ Long-term weight loss was calculated by taking the participants’ reported body mass 1 year ago or current body mass (the higher of the two), subtracting that figure from their historical maximum body mass, and then dividing by their historical maximum body mass and multiplying by 100. ^b^ Poor sleepers are those that sleep less than 7 h per night. Ideal sleepers are those that report sleeping for ≥7 h per night. ^c^ Inactive are those that accumulate less than 150 min per week of moderate- to vigorous-intensity aerobic physical activity. ^d^ Active are those that accumulate greater than or equal to 150 min per week of moderate- to vigorous-intensity aerobic physical activity. All models are adjusted for age, sex, race/ethnicity, education, marital status, household income and size, self-rated health status, smoking, and daily caloric intake. Each independent variable was entered into a separate regression model, and accelerometer wear time was adjusted for in the models where accelerometers were used (i.e., sedentary time and physical activity).

**Table 4:** Two-step regression analysis of joint associations of sleep, sedentary time, and physical activity with long-term weight loss success ^a^.

	Step 1—Logit Model		Step 2—GLM Model	
	OR (95% CI)	*p*	Coef. (SE)	*p*
Sleep + Sedentary				
Poor sleep ^b^ + high sedentary	Ref.		Ref.	
Poor sleep ^b^ + low sedentary	1.23 (0.79–1.90)	0.36	−0.07 (0.15)	0.64
Ideal sleep ^c^ + high sedentary	0.93 (0.67–1.31)	0.68	−0.17 (0.10)	0.10
Ideal sleep ^c^ + low sedentary	1.27 (0.84–1.92)	0.27	−0.14 (0.17)	0.39
Physical activity	1.31 (0.98–1.74)	0.07	−0.13 (0.08)	0.09
Sleep + Physical Activity				
Poor sleep ^b^ + inactive ^d^	Ref.		Ref.	
Poor sleep ^b^ + active ^e^	0.98 (0.64–1.49)	0.92	−0.18 (0.11)	0.12
Ideal sleep ^c^ + inactive ^d^	0.83 (0.61–1.12)	0.22	−0.15 (0.10)	0.14
Ideal sleep ^c^ + active ^e^	1.28 (0.88–1.86)	0.20	−0.25 (0.09)	0.01
Sedentary	1.32 (0.95–1.84)	0.10	−0.02 (0.12)	0.88
Physical Activity + Sedentary				
Inactive ^d^ + high sedentary	Ref.		Ref.	
Inactive ^d^ + low sedentary	1.61 (1.12–2.31)	0.01	−0.18 (0.09)	0.06
Active ^e^ + high sedentary	1.61 (1.10–2.38)	0.02	−0.06 (0.16)	0.71
Active ^e^ + low sedentary	1.55 (1.05–2.28)	0.03	−0.13 (0.12)	0.26
Sleep	0.96 (0.75–1.24)	0.78	−0.13 (0.08)	0.11
Sleep + Sedentary + Physical Activity				
None of the behaviors	Ref.		Ref.	
Any 1 of the behaviors	1.12 (0.65–1.94)	0.68	0.27 (0.16)	0.11
Any 2 of the behaviors	1.22 (0.71–2.10)	0.48	0.11 (0.16)	0.49
All 3 of the behaviors	1.65 (0.91–2.98)	0.10	0.01 (0.17)	0.96

Abbreviations: CI, confidence interval; Coef, coefficient; OR, odds ratio; P, *p*-value; Ref, reference group. Note: All models were adjusted for age, sex, race/ethnicity, marital status, education, household income and size, smoking status, self-rated health status, daily caloric intake, and accelerometer wear time. Models for any doublet pair of behaviors were adjusted for the third behavior. ^a^ Long-term weight loss was calculated by taking the participants’ reported body mass 1 year ago or current weight (the higher of the two), subtracting that figure from their historical maximum body mass, and then dividing by their historical maximum body mass and multiplying by 100. ^b^ Poor sleepers are those that sleep less than 7 h per night. ^c^ Ideal sleep is defined as sleeping for ≥7 h per night. ^d^ Active are those that accumulate greater than or equal to 150 min per week of moderate to vigorous aerobic physical activity. ^d^ Inactive are those that accumulate less than 150 min per week of moderate- to vigorous-intensity aerobic physical activity. ^e^ Active are those that accumulate greater than or equal to 150 min per week of moderate- to vigorous-intensity aerobic physical activity.

## Data Availability

Publicly available datasets were analyzed in this study. This data can be found here: https://wwwn.cdc.gov/nchs/nhanes/ContinuousNhanes/Default.aspx?BeginYear=2005 (accessed on 4 March 2021).

## Appendix

**Table A1:** Two-step regression analysis of joint associations of sleep, sedentary time, and physical activity with long-term weight loss success ^a^.

	Step 1—Logit Model			Step 2—GLM Model		
	Odds Ratio	(95% CI)	*p*	Coef.	(SE)	*p*
Sleep + Sedentary						
Poor sleep ^b^ + high sedentary	Ref.			Ref.		
Low sedentary	1.21	(0.74–1.99)	0.44	0.29	(0.01–0.58)	0.05
Ideal sleep ^c^	0.87	(0.51–1.47)	0.59	0.14	(−0.15–0.43)	0.36
Ideal sleep ^c^ + low sedentary	1.17	(0.65–2.11)	0.61	−0.32	(−0.66–0.02)	0.06
Physical activity	1.32	(0.99–1.75)	0.06	−0.15	(−0.31–0.00)	0.05
Sleep + Physical Activity						
Poor sleep ^b^ + inactive ^e^	Ref.			Ref.		
Active ^d^	1.00	(0.66–1.52)	0.99	0.00	(−0.41–0.42)	0.10
Ideal sleep ^c^	0.84	(0.62–1.13)	0.25	−0.18	(−0.48–0.12)	0.13
Ideal sleep ^c^ + active	1.55	(0.93–2.58)	0.10	0.07	(−0.22–0.35)	0.65
Sedentary	1.33	(0.94–1.89)	0.11	0.11	(−0.10–0.31)	0.30
Physical Activity + Sedentary						
Inactive ^e^ + high sedentary	Ref.			Ref.		
Low sedentary	1.58	(1.04–2.42)	0.03	0.16	(−0.09–0.42)	0.21
Active ^d^	1.80	(1.03–3.12)	0.04	−0.04	(−0.34–0.26)	0.80
Active ^d^ + low sedentary	0.67	(0.36–1.22)	0.19	−0.14	(−0.49–0.21)	0.43
Ideal sleep ^c^	0.98	(0.76–1.26)	0.85	−0.13	(−0.29–0.02)	0.09

Abbreviations: CI, confidence interval; Coef, coefficient; OR, odds ratio; P, *p*-value; Ref, reference group. Note: All models were adjusted for age, sex, race/ethnicity, marital status, education, household income and size, smoking status, self-rated health status, daily caloric intake, and accelerometer wear time. Models for any doublet pair of behaviors were adjusted for the third behavior. Computed models include interactions and the main effects of interaction terms. ^a^ Long-term weight loss was calculated by taking the participants’ reported body mass 1 year ago or current weight (the higher of the two), subtracting that figure from their historical maximum body mass, and then dividing by their historical maximum body mass and multiplying by 100. ^b^ Poor sleepers are those that sleep less than 7 h per night. ^c^ Ideal sleep is defined as sleeping for ≥7 h per night. ^d^ Active are those that accumulate greater than or equal to 150 min per week of moderate to vigorous aerobic physical activity. ^e^ Inactive are those that accumulate less than 150 min per week of moderate- to vigorous-intensity aerobic physical activity. ^e^ Active are those that accumulate greater than or equal to 150 min per week of moderate- to vigorous-intensity aerobic physical activity.

## References

[1] Nicklas J.M., Huskey K.W., Davis R.B., Wee C.C. (2012). Successful Weight Loss among Obese US Adults. Am. J. Prev. Med..

[2] Kraschnewski J.L., Boan J., Esposito J., Sherwood N.E., Lehman E.B., Kephart D.K., Sciamanna C.N. (2010). Long-term Weight Loss Maintenance in the United States. Int. J. Obes..

[3] Avenell A., Brown T.J., McGee M., Campbell M.K., Grant A.M., Broom J., Jung R., Smith W. (2004). What are the Long-term Benefits of Weight Reducing Diets in Adults? A Systematic Review of Randomized Controlled Trials. J. Hum. Nutr. Diet..

[4] Dombrowski S.U., Knittle. K., Avenell A., Araújo-Soares V., Sniehotta F.F. (2014). Long Term Maintenance of Weight Loss with Non-surgical Interventions in Obese Adults: Systematic Review and Meta-analyses of Randomised Controlled Trials. BMJ.

[5] MacLean P.S., Wing R.R., Davidson T., Epstein L., Goodpaster B., Hall K.D., Levin B.E., Perri M.G., Rolls B.J., Rosenbaum M. (2015). NIH working group report: Innovative research to improve maintenance of weight loss. Obesity.

[6] Knell G., Li Q., Gabriel K.P., Shuval K. (2018). Long-term weight loss and metabolic health in adults concerned with maintaining or losing weight: Findings from NHANES. Mayo Clin. Proc..

[7] Jensen M.D., Ryan D.H., Apovian C.M., Ard J.D., Comuzzie A.G., Donato K.A., Hu F.B., Hubbard V.S., Jakicic J.M., Kushner R.F. (2013). AHA/ACC/TOS guideline for the management of overweight and obesity in adults: A report of the American college of cardiology/American heart association task force on practice guidelines and the obesity society. Circulation.

[8] Moore J.B., Boesch C. (2019). Getting energy balance right in an obesogenic world. Proc. Nutr. Soc..

[9] Katz D.L. (2014). Perspective: Obesity is not a disease. Nature.

[10] Swift D.L., McGee J.E., Earnest C.P., Carlisle E., Nygard M., Johannsen N.M. (2018). The effects of exercise and physical activity on weight loss and maintenance. Prog. Cardiovasc. Dis..

[11] Wing R.R., Hill J.O. (2001). Successful weight loss maintenance. Annu. Rev. Nutr..

[12] Gilis-Januszewska A., Barengo N.C., Lindström J., Wójtowicz E., Acosta T., Tuomilehto J., Schwarz P.E., Piwońska-Solska B., Szybiński Z., Windak A. (2018). Predictors of Long Term Weight Loss Maintenance in Patients at High Risk of Type 2 Diabetes Participating in a Lifestyle Intervention Program in Primary Health Care: The DE-PLAN Study. PLoS ONE.

[13] Paixão C., Dias C.M., Jorge R., Carraça E.V., Yannakoulia M., de Zwaan M., Soini S., Hill J.O., Teixeira P.J., Santos I. (2020). Successful Weight Loss Maintenance: A Systematic Review of Weight Control Registries. Obes. Rev..

[14] Catenacci V.A., Pan Z., Thomas J.G., Ogden L.G., Roberts S.A., Wyatt H.R., Wing R.R., Hill J.O. (2014). Low/no calorie sweetened beverage consumption in the national weight control registry. Obesity.

[15] Grgic J., Dumuid D., Bengoechea E.G., Shrestha N., Bauman A., Olds T., Pedisic Z. (2018). Health Outcomes Associated with Reallocations of Time between Sleep, Sedentary Behaviour, and Physical Activity: A Systematic Scoping Review of Isotemporal Substitution Studies. Int. J. Behav. Nutr. Phys. Act..

[16] Melby C.L., Paris H.L., Sayer R.D., Bell C., Hill J.O. (2019). Increasing Energy Flux to Maintain Diet-Induced Weight Loss. Nutrients.

[17] Pronk N.P., Wing R.R. (1994). Physical Activity and Long-term Maintenance of Weight Loss. Obes. Res..

[18] Tremblay M.S., Aubert S., Barnes J.D., Saunders T.J., Carson V., Latimer-Cheung A.E., Chastin S.F., Altenburg T.M., Chinapaw M.J. (2017). Sedentary Behavior Research Network (SBRN)–Terminology Consensus Project Process and Outcome. Int. J. Behav. Nutr. Phys. Act..

[19] Kerrigan S.G., Call C., Schaumberg K., Forman E., Butryn M.L. (2018). Associations between change in sedentary behavior and outcome in standard behavioral weight loss treatment. Transl. Behav. Med..

[20] Rees-Punia E., Guinter M.A., Gapstur S.M., Wang Y., Patel A.V. (2021). Composition of time in movement behaviors and weight change in latinx, black and white participants. PLoS ONE.

[21] Ostendorf D.M., Lyden K., Pan Z., Wyatt H.R., Hill J.O., Melanson E.L., Catenacci V.A. (2018). Objectively measured physical activity and sedentary behavior in successful weight loss maintainers. Obesity.

[22] Clamp L.D., Hume D.J., Lambert E.V., Kroff J. (2018). Successful and unsuccessful weight-loss maintainers: Strategies to counteract metabolic compensation following weight loss. J. Nutr. Sci..

[23] King W.C., Belle S.H., Hinerman A.S., Mitchell J.E., Steffen K.J., Courcoulas A.P. (2020). Patient behaviors and characteristics related to weight regain after roux-en-Y gastric bypass: A multicenter prospective cohort study. Ann. Surg..

[24] Pellegrini C.A., Song J., Chang R.W., Semanik P.A., Lee J., Ehrlich-Jones L., Pinto D., Dunlop D.D. (2016). Change in physical activity and sedentary time associated with 2-year weight loss in obese adults with osteoarthritis. J. Phys. Act. Health.

[25] Wakaba K., Sasai H., Nakata Y. (2020). Associations of objectively measured physical activity and sleep with weight loss maintenance: A preliminary study of Japanese adults. Behav. Sci..

[26] Larsen S.C., Horgan G., Mikkelsen M.L.K., Palmeira A.L., Scott S., Duarte C., Santos I., Encantado J., O’Driscoll R., Turicchi J. (2020). Consistent sleep onset and maintenance of body weight after weight loss: An analysis of data from the NoHoW trial. PLoS Med..

[27] Curtin L.R., Mohadjer L.K., Dohrmann S.M., Montaquila J.M., Kruszan-Moran D., Mirel L.B., Carroll M.D., Hirsch R., Schober S., Johnson C.L. (2012). The National Health and Nutrition Examination Survey: Sample Design, 1999–2006. Vital Health Stat. Ser. 2 Data Eval. Methods Res..

[28] Troiano R.P., McClain J.J., Brychta R.J., Chen K.Y. (2014). Evolution of Accelerometer Methods for Physical Activity Research. Br. J. Sports Med..

[29] Tudor-Locke C., Camhi S.M., Troiano R.P. (2012). A Catalog of Rules, Variables, and Definitions Applied to Accelerometer Data in the National Health and Nutrition Examination Survey, 2003–2006. Prev. Chronic Dis..

[30] Troiano R.P., Berrigan D., Dodd K.W., Masse L.C., Tilert T., McDowell M. (2008). Physical Activity in the United States Measured by Accelerometer. Med. Sci. Sports Exerc..

[31] NHANES 2003–2004 Physical Activity Monitor Data Documentation, Codebook, and Frequencies. http://www.cdc.gov/nchs/nhanes/nhanes2003-2004/PAXRAW_.

[32] Freedson P.S., Melanson E., Sirard J. (1998). Calibration of the Computer Science and Applications, Inc. Accelerometer. Med. Sci. Sports Exerc..

[33] Piercy K.L., Troiano R.P., Ballard R.M., Carlson S.A., Fulton J.E., Galuska D.A., George S.M., Olson R.D. (2018). The Physical Activty Guidelines for Americans. JAMA.

[34] Kushi L.H., Doyle C., McCullough M., Rock C.L., Demark-Wahnefried W., Bandera E.V., Gapstur S., Patel A.V., Andrews K., Gansler T. (2012). American Cancer Society Guidelines on Nutrition and Physical Activity for Cancer Prevention: Reducing the Risk of Cancer with Healthy Food Choices and Physical Activity. CA A Cancer J. Clin..

[35] Katzmarzyk P.T., Powell K.E., Jakicic J.M., Troiano R.P., Piercy K., Tennant B. (2019). Committee PAGA: Sedentary Behavior and Health: Update from the 2018 Physical Activity Guidelines Advisory Committee. Med. Sci. Sports Exerc..

[36] Chasens E.R., Ratcliffe S.J., Weaver T.E. (2009). Development of the FOSQ-10: A Short Version of the Functional Outcomes of Sleep Questionnaire. Sleep.

[37] Weaver T.E., Laizner A.M., Evans L.K., Maislin G., Chugh D.K., Lyon K., Smith P.L., Schwartz A.R., Redline S., Pack A.I. (1997). An Instrument to Measure Functional Status Outcomes for Disorders of Excessive Sleepiness. Sleep.

[38] Panel C.C., Watson N.F., Badr M.S., Belenky G., Bliwise D.L., Buxton O.M., Buysse D., Dinges D.F., Gangwisch J., Grandner M.A. (2015). Recommended Amount of Sleep for a Healthy Adult: A Joint Consensus Statement of the American Academy of Sleep Medicine and Sleep Research Society. J. Clin. Sleep Med..

[39] Stommel M., Osier N. (2013). Temporal changes in bias of body mass index scores based on self-reported height and weight. Int. J. Obes..

[40] Casey V.A., Dwyer J.T., Berkey C.S., Coleman K.A., Gardner J., Valadian I. (1991). Long-Term Memory of Body Weight and Past Weight Satisfaction: A Longitudinal Follow-up Study. Am. J. Clin. Nutr..

[41] Benowitz N.L., Bernert J.T., Caraballo R.S., Holiday D.B., Wang J. (2009). Optimal serum cotinine levels for distinguishing cigarette smokers and nonsmokers within different racial/ethnic groups in the United States between 1999 and 2004. Am. J. Epidemiol..

[42] Santos I., Vieira P.N., Silva M.N., Sardinha L.B., Teixeira P.J. (2017). Weight Control Behaviors of Highly Successful Weight Loss Maintainers: The Portuguese Weight Control Registry. J. Behav. Med..

[43] Ostendorf D.M., Caldwell A.E., Creasy S.A., Pan Z., Lyden K., Bergouignan A., MacLean P.S., Wyatt H.R., Hill J.O., Melanson E.L. (2019). Physical activity energy expenditure and total daily energy expenditure in successful weight loss maintainers. Obesity.

[44] Ross K.M., Thomas J.G., Wing R.R. (2016). Successful weight loss maintenance associated with morning chronotype and better sleep quality. J. Behav. Med..

[45] Chaput J.P., Saunders T., Carson V. (2017). Interactions between sleep, movement and other non-movement behaviours in the pathogenesis of childhood obesity. Obes. Rev..

[46] Rosenberger M.E., Buman M.P., Troiano R.P., Grandner M.A., Buchner D.M., Haskell W.L. (2019). The 24-hour Activity Cycle: A New Paradigm for Physical Activity. Med Sci Sports Exerc..

[47] Shuval K., Leonard T., Drope J., Katz D.L., Patel A.V., Maitin-Shepard M., Amir O., Grinstein A. (2017). Physical Activity Counseling in Primary Care: Insights from Public Health and Behavioral Economics. CA Cancer J. Clin..

[48] Curry S.J., Krist A.H., Owens D.K., Barry M.J., Caughey A.B., Davidson K.W., Doubeni C.A., Epling J.W., Grossman D.C., Kemper A.R. (2018). Behavioral Weight Loss Interventions to Prevent Obesity-related Morbidity and Mortality in Adults: US Preventive Services Task Force Recommendation Statement. JAMA.

[49] Rothman K.J., Greenland S., Lash T.L. (2008). Modern Epidemiology.

